# Microstructural Evolution and Mechanical Properties of Pure Aluminum upon Multi-Pass Caliber Rolling

**DOI:** 10.3390/ma15031206

**Published:** 2022-02-05

**Authors:** Shulong Guo, Hui Yu, Zhifeng Wang, Wei Yu, Weili Cheng, Lixin Huang, Chunhai Liu, Fuxing Yin, Weimin Zhao, Chunling Qin

**Affiliations:** 1 School of Materials Science and Engineering, Hebei University of Technology, Tianjin 300401, China; guoshulong666@163.com (S.G.); yinfuxing@hebut.edu.cn (F.Y.); wmzhao@hebut.edu.cn (W.Z.); 2 Hefei Nova Advanced Materials Co., Ltd., Hefei 230000, China; yuwei52213@163.com; 3 School of Materials Science and Engineering, Hefei University of Technology, Hefei 230009, China; 4 School of Materials Science and Engineering, Taiyuan University of Technology, Taiyuan 030024, China; chengweili7@126.com; 5 CITIC Dicastal Co., Ltd., Qinhuangdao 066011, China; lxhuanglucky@163.com (L.H.); liuchunhai@dicastal.com (C.L.); 6 Institute of New Materials, Guangdong Academy of Sciences, Guangzhou 510651, China

**Keywords:** pure Al, caliber rolling, microstructure, texture, mechanical property

## Abstract

The paper presents the microstructure and mechanical property of pure aluminum (Al) fabricated by multi-pass caliber rolling at room temperature. The finite element modeling (FEM) simulation was performed to explore the changes in rolling force, effective stress and strain, and temperature under various rolling passes. As the number of rolling passes increased, the overall temperature, effective stress, and strain gradually increased, while the maximum rolling force decreased. In addition, due to the dynamic recrystallization (DRX), the average grain size reduced from 1 mm to 14 µm with the increase in rolling passes. The dislocation density increased and it gradually evolved into the high-angle grain boundaries (HAGBs). Moreover, the initial cubic texture rotated to the brass component and finally changed to a mixture of Cube and Brass types. The highest tensile yield strength (TYS), ultimate tensile strength (UTS) and elongation (El.) of caliber rolled pure Al (116 MPa, 135 MPa, and 17%, respectively) can be achieved after 13 rolling passes, which mainly attributed to grain refinement.

## 1. Introduction

Nowadays, the researchers in automotive and aerospace fields are eager to develop high-performance structural metals, in which lightweight alloys become an important choice for such applications [1]. Aluminum (Al) and its alloys are widely used due to their high strength, good formability, and corrosion resistance [2,3,4,5,6]. To meet the increasing toughness requirements of structural materials, severe plastic deformation (SPD) technology is introduced to greatly improve the strength of materials [7,8,9,10]. Usually, the SPD refers to applying a large plastic strain at a certain temperature, changing the microstructure of the material to improve its mechanical properties, and finally obtaining an ultrafine-grained (UFGed) microstructure [11]. The grain size of UFGed material can reach even the nanometer level, which in turn, fine-grain strengthening can be obtained. After SPD, continuous dynamic recrystallization (CDRX) occurs, resulting in a mosaic-like structure or a cell block construction retarded by a dislocation wall [12]. As the deformation progresses, the dislocation density increases and it gradually evolves into the high-angle grain boundaries (HAGBs) [13]. Thus, the control of grain boundary (GB) seems one of the effective methods for developing materials with excellent mechanical properties.

Mustafa et al. [14] explored the rotary swaging (RS) deformation of pure Al and found that when the true strain was 3, the tensile yield strength (TYS), and ultimate tensile strength (UTS) are 8 and 2 times that of the non-deformed sample, respectively. The RS was presented to lead to a significant decrease in grain size and introduced about 70% Low-angle grain boundaries (LAGBs). Naoya et al. [15] studied the accumulative roll bonding (ARB) of pure Al and obtained TYS of 114 MPa after 6 passes, the enhanced mechanical property resulted from the synergy effect of both grain boundary strengthening and dislocation strengthening. Wacek et al. [16] investigated extruded pure Al under low temperature using liquid nitrogen, the grain size can be reduced to 400 nm, and the TYS under this circumstance was increased to 168 MPa (or 56 Hv). Due to the cost and operability of the experiment, it is not applicable in mass production. Soroosh et al. [17] used high-pressure torsion (HPT) technology to decrease grain size and increase the misorientation to form HAGBs of pure Al, when the temperature and the equivalent strain were 773 K and 99, the grain size decreased to about 1 μm and the maximum hardness increased to 54 Hv, respectively. According to Chrominski et al. [18], 8 passes of equal channel angular pressing (ECAP) remarkably refined the grain size of pure Al sheet below 500 nm with about 80% HAGBs, which also showed a high hardness value of 58 Hv. Although the above-mentioned SPD technologies can greatly improve the mechanical properties of pure Al, it also presents some disadvantages, such as complicated operation, harsh experimental conditions with high costs, and unsuitable for large-scale production in actual. So, the driving force of this study is to develop a new approach to fabricating bulk pure Al with high strength.

Recently, one SPD, called caliber rolling, showed advantages for mass production of bulk materials with an ultra-fine grain (UFG) in not only traditional steel but also non-ferrous metals (i.e., Al [19], Mg [20] alloys). For instance, the caliber rolled Al-Si-Mg-Fe alloy shows Fe-intermetallic compounds refined to about 200 nm, given UTS and elongation (El.) were refined to about 360 MPa and 25%, respectively. Moreover, when the extruded Mg-3Al-1Zn-0.2Mn (wt.%) alloy caliber rolled for 18 passes, its TYS exceeded 400 MPa, besides, the weakening of the texture caused by the shear deformation also reduced the yield asymmetry. In addition, the Compressive yield strength at room temperature over 500 MPa can be achieved by 6 passes caliber rolling in Mg-5Y-2.5Zn (at.%) alloy. It is easy to conclude that GB control plays an important role in caliber rolling and bring benefit to the strength and toughness of metals [21,22]. Up to now, there is a rare report about caliber rolling of pure Al, especially the relationship between microstructure and mechanical property of caliber rolled pure Al need to be clarified and better understood. The initial step to investigating the DRX involved deformation behavior is to figure out the effects of processing parameters on temperature, strain, and stress about Al and its alloys. Thus, FEM sounds like a reasonable method to understand such information in detail. Djavanroodi et al. [23] analyzed ECAPed commercial pure Al using FEM and found the origin of high punch/press pressure came from the increasing strain accumulated. In addition, other Al and Al alloys research [24,25] represented the prediction of stress and stress during deformation by FEM analysis agreed well with actual experimental results.

Thus, the objective of the presented study is to investigate the microstructure, texture evolution, and mechanical properties of pure Al using multi-pass caliber rolling at room temperature, especially focusing on the influence of rolling pass. We hope this study can not only give more depth thinking about caliber rolled pure Al with high performance but also provide a solution for mass-production of bulk Al and its alloy to extend their application in near future.

## 2. Materials and Methods

**Figure 1:**
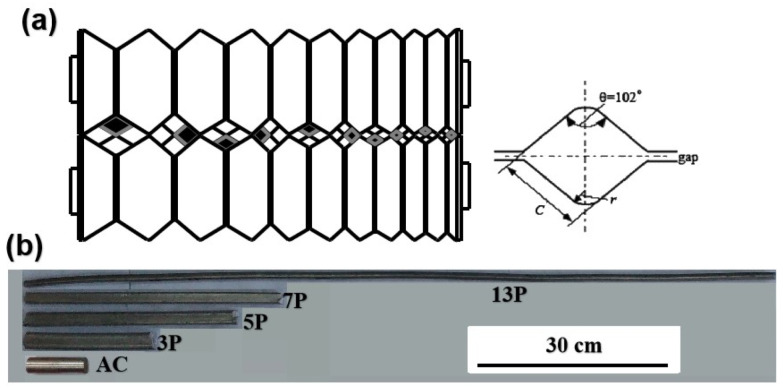
(**a**) Schematic illustrations of groove shape used in this study; (**b**) As-cast and As-rolled bars after various passes. (P: pass).

The pure Al (nominal composition in wt.%: Si, 0.45%; Zn, 0.01%; Cu, 0.05% ~ 0.20%; Mn, 0.03%; Fe, 0.035%; Al, 99%; purchased from Furui Metal Products Co., Ltd., Shanghai, China; AA1100) was selected and caliber rolled into a bar with a size of Φ35 × 100 mm at room temperature. The dimensions of each rolling groove were illustrated in Figure 1a and Table 1. After the sample rolled for each pass, the bar was rotated 90° before the next pass in order to reduce the unevenness of the reduction. At the final pass, the rolling was repeated to ensure the size of the bar and the corresponding temperature were recorded. In the present study, the 3, 5, 7, and 13 passes rolling with an area reduction of about 18% per pass were carried out and the rolled bars with sound surface were shown in Figure 1b. Dimension of groove and roll gap for different passes were shown in Table 1. Infrared thermometer (CENTER-350, SHUANGXU, Shanghai, China) was used to obtain the surface temperature of different passes of rolled bars. The temperature measurement position was selected to conduct five tests in the middle of the rolled bar and we took the average value as the final result.

In addition, the FEM analysis using DEFORMTM 3D software (Scientific Forming Technologies Corporation, Columbus, OH, USA) was used to predict the feasibility of caliber rolling of pure Al at room temperature. A total of 13 rolls models were established by Pro/E and imported. Since the composition of AA1100 is basically the same as that of pure Al in this paper, the default pure Al database in software was used, and the meshing of the bar was about 32,000 tetrahedral. The rolling speed was set to 0.2 m/s, and the number of steps and step length were set to 60 and 0.05, respectively. The FEM Simulation Parameters of pure Al were illustrated in Table 2.

The specimen for electron backscattered diffraction (EBSD) inspection were ground with sandpaper and mechanically polished with Al_2_O_3_ paste, then the electrolytic polishing was performed using ASCII electrolyte (50 mL isopropanol + 20.75 g sodium thiocyanate + 37.5 g citric acid + 400 mL ethanol + 7.5 mL perchloric acid + 9 mL distilled water + 5 g hydroxyquinoline) at −15 °C for 3~5 min. The EBSD analysis was carried out with a field emission scanning electron microscope (FESEM, SU-6600, Hitachi High-Tech Group, Tokyo, Japan), and EDAX TSL OIM7 (Philadelphia, USA) software was used for data collection and characterization, the measured datasets with CI > 0.7 were used for examination, characterizing information such as crystal grain morphology, orientation, and texture. Wire cut electrical discharge machining (WEDM, HENGSONG, Shenyang, China) was used to cut sample slice and ground into 100 μm, then punched to Φ3 mm disc. The transmission electron microscope (TEM, Tecnai G^2^ 20, FEI Company, Hillsboro, USA) samples were prepared using Jet-polisher with an electrolytic of 80% CH_3_OH and 20% HNO_3_ at a current of 10 mA and temperature of −20 °C, respectively. Then the TEM observation of the sample was performed on Tecnai G^2^ 20. The fracture morphologies were carried out using a JEOL JSM-7000F scanning electron microscope (SEM, JEOL, Tokyo, Japan). The microhardness was tested using a 402SXV digital microhardness (Shanghai, China) instrument with a loading of 98 mN and a dwell time of 15 s. In addition, dog-bone-shaped specimens with a gage dimension of Φ8 mm × 60 mm were used for the tensile tests. The tensile tests were conducted using an electro-universal mechanical testing machine (SUNS-UTM5105X, SHENZHEN SUNS TECHNOLOGY STOCK CO., LTD., Shenzhen, China) at room temperature, with an initial strain rate of 0.001 s^−1^ along the rolling direction (RD). All samples were tested 3 times for avoiding inaccuracy. After the tensile tests, the fracture morphologies of the caliber-rolled sample were characterized by SEM and compared with those of the as-cast sample.

## 3. Results and Discussion

**Figure 2:**
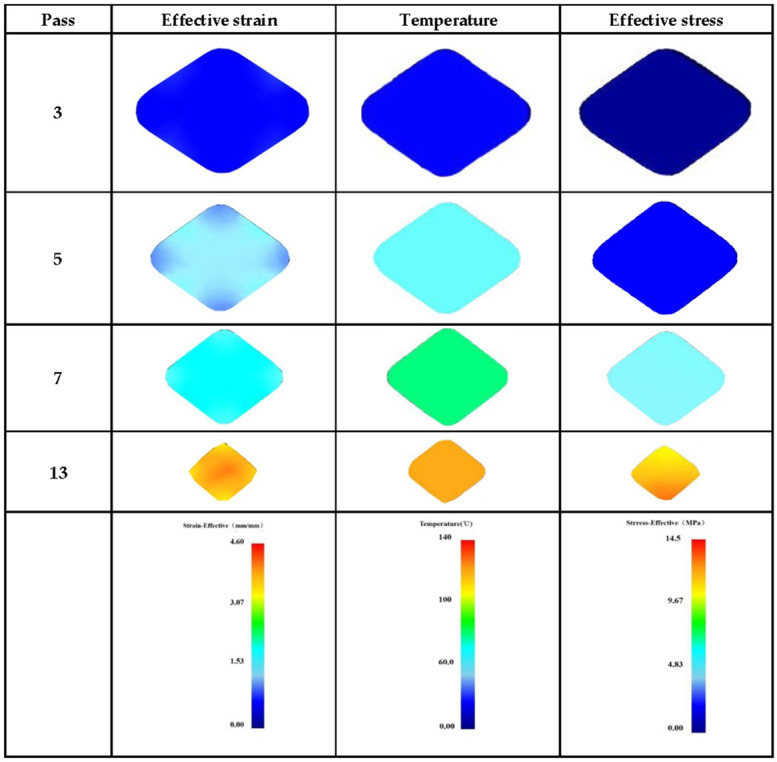
Distribution of effective strain, temperature, and effective stress in different passes by FEM simulation results, respectively.

Figure 2 shows the FEM simulation results (i.e., effective strain, temperature, and effective stress) upon different rolling passes. It can be seen that as the rolling pass increased, the effective strain gradually accumulated. For example, the cumulative deformation after 3 passes and 13 passes were 45.23% and 92.48%, and the effective strain increased from 0.622 to 3.61, respectively. In addition, the effective strain at the core and end face area was higher than that edge part of the rolled bar, which is due to the existence of the roll gap giving more freedom partially.

**Figure 3:**
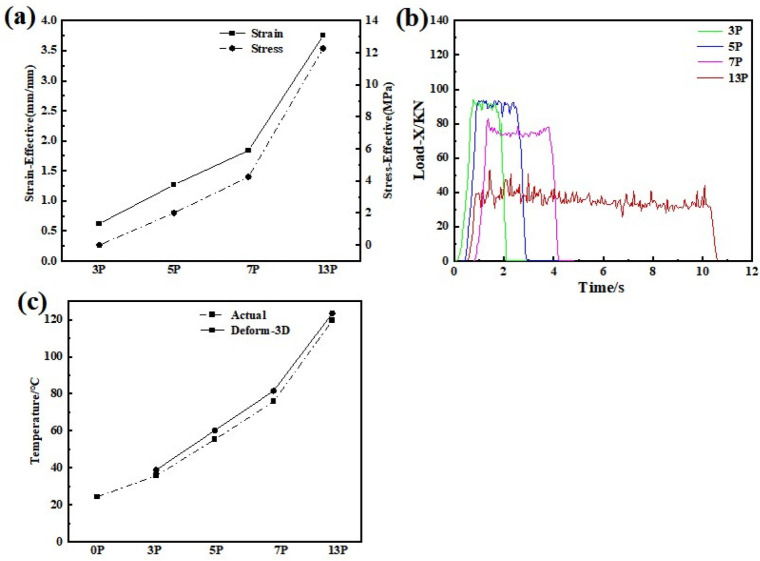
(**a**) Effective stress and effective strain curves of different rolling passes; (**b**) rolling force in terms of time under various rolling passes; (**c**) temperature difference between FEM prediction and actual measurement.

Effective stress is an important term for the plastic deformation of materials and can be understood as the comprehensive effect of the stress deflection tensor. As shown in Figure 2 and Figure 3a, in the case of 3 passes, the effective stress tended to be 0. As the rolling passes increased, the equivalent stress increased little by little. This value reached about 13 MPa after 13 passes.

Moreover, Figure 3b showed the diversity of rolling force in different rolling passes. Three stages can be clearly distinguished by the input pressure, (i) the rolling force increased rapidly called the rolling bite stage; (ii) the rolling force fluctuated slightly and maintained a stable stage; (iii) the force quickly downed to zero mean rolling almost end. There is no obvious difference in the maximum rolling force between 3 and 5 passes, but the gap became larger as roll passes increased. For instance, the maximum rolling force of 13 passes was only half of 3 passes. This phenomenon can be explained by the increase in temperature as the number of rolling passes increases. The higher temperature introduced, the easier deform occurred, which in turn, the rolling force decreased accordingly. This change can also be verified in Figure 2 and Figure 3c, which gives an example of deformation heat generated with rolling passes increased. It can be found that larger area reduction resulted in higher temperature, i.e., in the case of 3-pass rolling showing about 40 °C, while it grew to 120 °C after 13-pass rolling. Furthermore, compared with the simulation results, the measured temperature was a little bit lower, which is due to a slight difference between the roller and the sample. In particular, the error between the simulation result and the actual temperature was very small, which implied that the developed FEM model showed acceptable validation.

**Figure 4:**
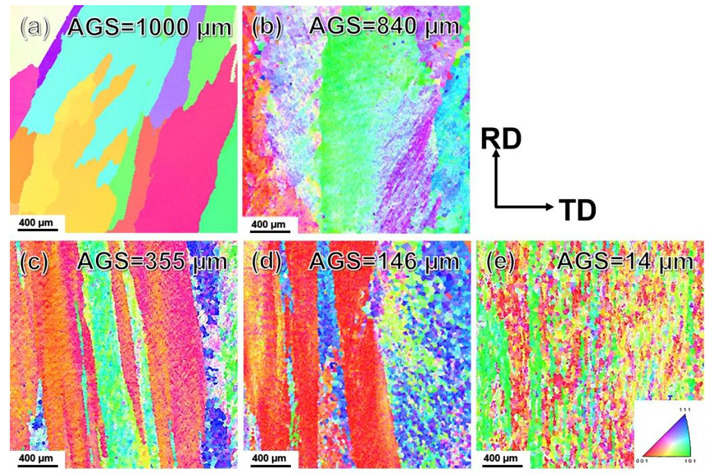
The inverse pole figure maps of as-cast and multi-pass caliber rolled pure Al: (**a**) as-cast; (**b**) 3P; (**c**) 5P; (**d**) 7P; (**e**) 13P. (AGS—average grain size).

**Figure 5:**
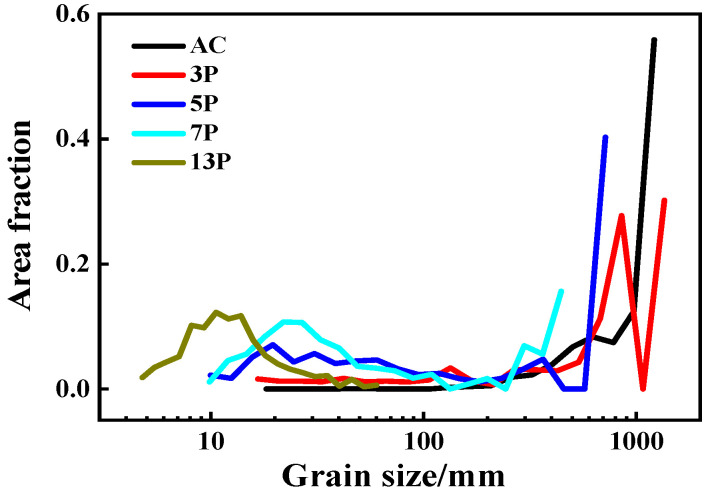
Statistics of the grain size distribution of as-cast and multi-pass caliber rolled pure Al.

Figure 4 showed the EBSD analysis results of as-cast and as rolled pure Al under different passes. The average grain size of the caliber rolled pure Al was smaller than that of the as-cast counterpart. Besides, as rolling passes increased, the grains were significantly refined. Figure 4a showed the original coarse columnar crystals in as-cast pure Al and the average grain size (AGS) was about 1000 µm. When rolling for 3 passes (see Figure 4b), the original coarse grains were arranged parallel to the RD. Thanks to the shear strain by rolling, a series of sub-crystal bands with LAGBs were generated in the parent coarse grain. As the amount of deformation intensified, the sub-grains were gradually refined and some CDRX grains with AGS of about 840 µm began to appear at random. As shown in Figure 4c for 5-pass rolling, the grains were significantly refined to about 355 µm. This grain refinement mainly owing to accumulated strain (see Figure 2). With the strain increased, the coarse columnar grains became slenderer and changed their direction parallel to the RD. Besides, when the deformation level was attained to a certain extent, the HAGB and equiaxed fine-grained structure were formed. From Figure 4d,e, the AGS was further reduced to 14 μm for 7 passes and even 14 μm for 13 passes, respectively. Moreover, the original coarse grains almost disappeared gradually. The elongated equiaxed crystals with different crystal orientations replaced the columnar crystals. CDRX occurred during the entire rolling. Figure 5 presented the grain size distribution of the pure Al in as-cast state and as-rolled conditions. The same trend can be seen evidently. In short, as the number of rolling passes increased, the grain size decreased, and a more homogeneous microstructure can be obtained.

**Figure 6:**
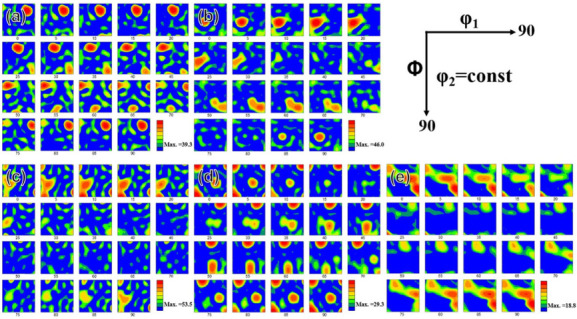
ODF maps of pure Al in (**a**) as-cast and (**b**–**e**) 3P, 5P, 7P and 13P caliber rolling.

Generally, the stacking fault energy (SFE) determines the mechanism of plastic deformation in SPD. High SFE materials (such as Al) are deformed by {111}<110> slip at room temperature. However, in low-SFE fcc materials, {111}<112> twinning will contribute a greater degree of deformation. In briefly, for high SFE materials, copper-type texture {100}<110> dominates; for low SFE materials, brass-type texture {112}<110> dominates [19,26]. Figure 6 showed the orientation distribution function (ODF) mapping of as-cast and as rolled pure Al. As shown in Figure 6a, the texture type of as-cast pure Al was rotated cubic texture {001}<110>. When rolling for 3 passes (see Figure 6b), the texture type changed to Brass (B component) {110}<112>. And it did not change in Figure 6c after 5 passes. In the case of 7 passes rolling, a recrystallized cubic texture Cube{001}<100> appeared, which consisted of the previously B component {110}<112>, as can be seen in Figure 6d. The ODF map of 13 passes specimen (see Figure 6e) showed a little different compared with Figure 6d but was still composed of recrystallized cubic texture Cube{001}<100> and Brass{110}<112>. These results also were consistent with other reports [27,28].

**Figure 7:**
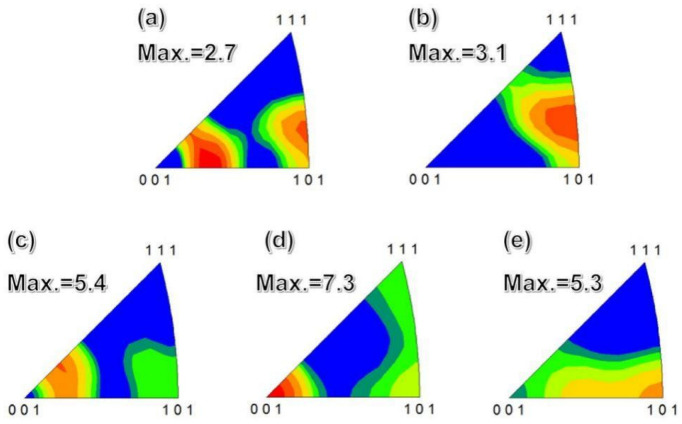
IPF of as-cast and multi-pass caliber rolled pure Al: (**a**) as-cast; (**b**) 3P; (**c**) 5P; (**d**) 7P and (**e**) 13P.

The inverse pole figures (IPF) of pure Al in different states as illustrated in Figure 7a–e. It can be found that as the number of rolling passes increased, the texture intensity gradually strengthened. For example, the IPF of as-cast and rolled 3 passes, the maximum intensity of texture was 3.1 mud and 5.4 mud respectively. After 7 rolling passes, it can be seen from Figure 7c that the maximum intensity of texture reached 7.3 mud. When the amount of deformation increased to a certain extent, the intensity of texture decreased instead. As shown in Figure 7e, during 13 passes of rolling, the maximum intensity of texture was reduced to 5.3 mud. The strong <001> texture and the weak <111> fiber texture are the typical texture of Al and its alloys during plastic deformation. As the number of rolling passes increased, the <101> texture (see Figure 7a,b) was replaced by the strong <001> texture (see Figure 7c,d), which shown strengthen the texture intensity by increasing of rolling passes. Then weak <111> fiber texture (see Figure 7e) was subsequently formed and decreased the intensity of texture. Complete recrystallization occurred inside the structure and the orientation of the recrystallized grains was more randomized so that the intensity of texture was weakened.

**Figure 8:**
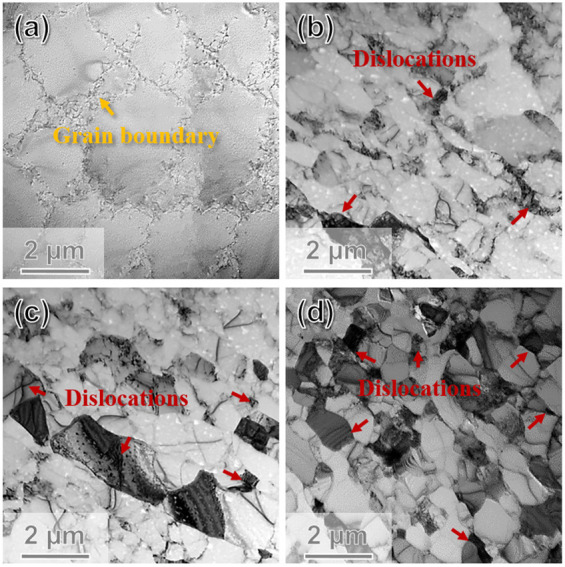
TEM bright-field images of (**a**) as-cast and (**b**–**d**) 5P, 7P, and 13P caliber rolled pure Al.

To further explore the grain refinement mechanism and DRX behavior, a TEM analysis was performed. The bright-field TEM images were displayed in Figure 8a–d. The TEM result of the 3-pass rolling sample showed a little bit of nuance with the 5-pass rolling one, so the 3 passes caliber rolled results were omitted here. Clearly, the grain size gradually decreased with rolling passes increases, which is also consistent with the EBSD results as shown in Figure 2. The coarse grains were easily observed in as-cast pure Al (see Figure 8a), and the matrix looked much cleaner. After rolling for 5 passes, as shown in Figure 8b, the dislocation began to gather at the grain boundaries and a dislocation tangle can be found. Besides, the grain size was also reduced a lot. When the rolling was continued, the dislocation propagated and formed new sub-grain boundaries. In general, the rolled bar is subjected to shear stress and dislocations will move along a certain slip. The original HAGBs have an obstructive effect on the motion of dislocations, making them obstructed at GB. Step by step, the interaction of dislocations will generate dislocation cells [16,19]. Figure 8c showed the density of dislocation increased and more and more dislocation were blocked at GBs. Usually, the sub-grains, as well as dislocations, might have contributed to the nuclei of recrystallization. Few nuclei had grown to grains, which meant CDRX occurred. Thus, the grain size was further reduced. After 13-pass rolling, as demonstrated in Figure 8d, high-density dislocation tangles mainly exist around the cell, forming the HAGBs via CDRX, and the grain size was greatly reduced, which should contribute to the high performance of caliber rolled pure Al.

**Figure 9:**
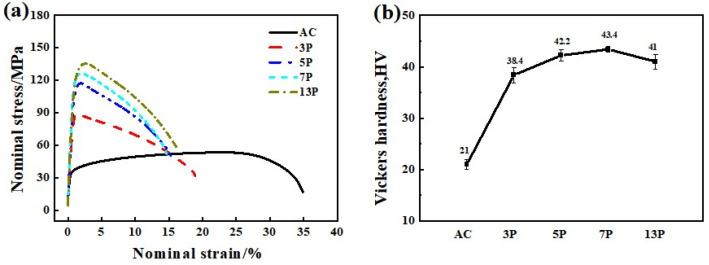
Typical tensile engineering stress-strain curves (**a**) and microhardness profile (**b**) of as-cast and multi-pass caliber rolled pure Al.

The mechanical properties including strain-stress curves and microhardness of as-cast and rolled pure Al was summarized in Figure 9. The TYS and UTS of as-cast Al were 32 MPa and 52 MPa showing a Vickers hardness of 21 Hv. Pure Al changed from “soft” to “hard” after 3 or 5-pass rolling and became “harder” for 7 passes rolling. Finally, the TYS, UTS, and hardness reached 115 MPa, 136 MPa, and 43 Hv after 13-pass rolling finished. Mechanical properties of as-cast and multi-pass caliber rolled pure Al was shown in Table 3. The high strength of caliber rolled pure Al was mainly due to the grain refinement of the DRXed grains (see Figure 4e and Figure 8d). In addition, the orientation of the new recrystallized grains was more randomized (see Figure 4d–e and Figure 7d–e), the texture weakened maybe take responsibility for the decrease in strength and hardness for the 13-pass caliber rolling specimen.

**Figure 10:**
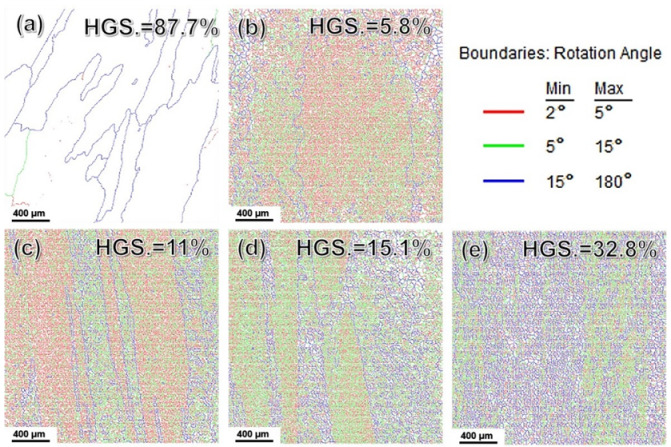
GBM of (**a**) as-cast and (**b**–**e**) 3P, 5P, 7P, and 13P caliber rolled pure Al, respectively.

**Figure 11:**
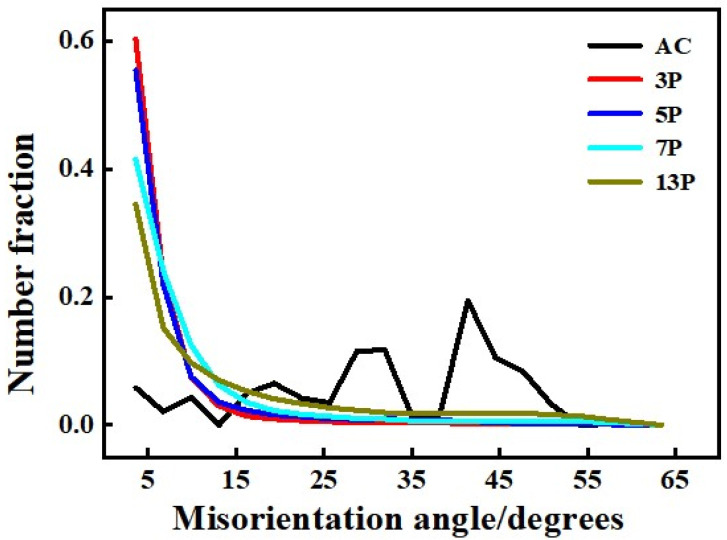
Misorientation distribution of as-cast and caliber rolled pure Al.

In order to further understand the effect of refined grain on the mechanical property of pure Al in different states, the grain boundary map (GBM) analysis was carried out, as shown in Figure 10a–e. The legend indicated various GB types, i.e., the red line for 2°~5°, green line for 5°~15°, and blue line for 15°~180°. The volume fraction (Vf) of HAGBs was also given in the upper right corner of each picture. It can be found that there were a large number of original HAGBs in as-cast pure Al which accounted for ~88% (see Figure 10a). The proportion of the HAGBs in 3-pass rolled samples was greatly reduced, only 5.8%. Since then, the amount of HAGBs became larger and larger as the number of rolling passes increased, as shown in Figure 10c,d, i.e., 11% for 5 passes and 15% for 7 passes caliber rolling, respectively. When 13-pass rolling was conducted, the Vf of HAGBs reached about 33% (see Figure 10e). These results were consistent with the TEM observation in Figure 8. The statistics of GB misorientation of all samples were listed in Figure 11. As mentioned above, a large number of dislocations were distributed at GB. The original coarse grains were divided into multiple smaller regions by geometrically necessary dislocations (GNDs), forming dislocation cells and becoming thicker and more regular. The formation of sub-grains led to a significant reduction in the proportion of the original HAGBs in as-rolled samples compared to the as-cast counterpart. As the deformation moved forward, the sub-grain boundaries would change to HAGBs, resulting in the Vf of HAGBs increasing again, and it was agreed well with previous studies [11,19].

According to related literature [29], the hardness value and grain size of the material satisfy the following equation:

(1) $${Hv=Hv0+K}_{HV}d^{-1/2}$$

where *Hv* is Vickers hardness, *Hv*_0_ is the hardness when the grain size is infinite, *K_HV_* is a constant and *d* is the average grain diameter.

Similarly, the relationship between strength and grain size can be also described by the classic Hall-Petch relationship [30]:

(2) $${\sigma =\sigma }_{0}{\;\pm \;K}^{-1/2}$$

where *σ* is yield strength, *σ*_0_ is the yield strength of a single crystal, *K* is constant, and *d* is the average grain size.

Taking Equation (1), Equation (2), and Figure 9 into account, the size of the grains significantly affected the strength and hardness of the materials. Smaller grain sizes brought higher hardness and strength. This is the primary reason for the mechanical property enhancement of multi-pass caliber rolling specimens.

**Figure 12:**
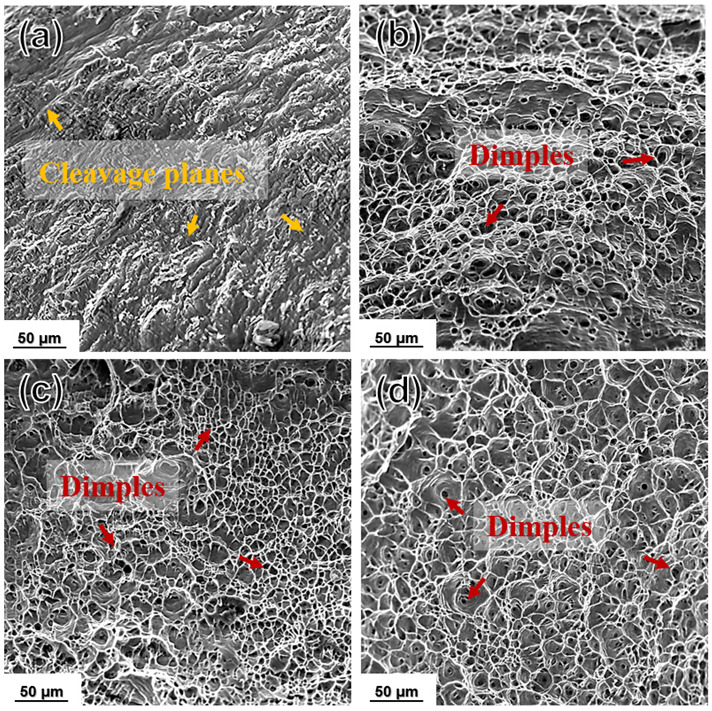
Typical SEM fracture morphology of (**a**) as-cast and (**b**–**d**) 5P, 7P, and 13P caliber rolled pure Al, respectively.

Figure 12 showed the typical SEM fracture surface of as-cast and as-rolled samples after the tensile test. All specimens showed a ductile characteristic, and the as-rolled sample with plenty of dimples can be found readily. In addition, the dimples became more uniform and finer as the number of rolling passes increased, which could be attributed to the fine grain size and homogenized microstructure.

Table 4 consists of different SPD technique routines. Among them, pure Al rolled by caliber rolling at room temperature exhibited good balance for high strength and ductility. In summary, caliber rolling has been successfully proposed as a prospective method to produce large-scale bulk light metals with high performance.

## 4. Conclusions

By utilization of multi-pass caliber rolling technique, high-performance pure Al showing TYS of 116 MPa, UTS of 135 MPa, and El. of 17%, respectively, is successfully fabricated. The enhancement of mechanical property was mainly attributed to the grain refinement by CDRX. In addition, FEM simulation results are consistent with the actual ones, which can give more precise predictions and insightful details during the rolling. The texture is a typical deformed one and the intensity firstly increased and then decreased as the rolling pass increased. The caliber rolling at room temperature can effectively refine the grains of pure Al from 1 mm to 14 µm, and the rolled bar showed sound surface finishing. In a word, the caliber rolling technology can not only significantly refine the grains, but also improve related mechanical properties, showing a prospective method for mass-production of large-size bulk Al and its alloys in near future.

## Figures and Tables

**Table 1:** Dimension of groove and roll gap for different passes.

Pass	c/mm	R	Gap/mm	Cumulative Reduction in Area	Cumulative Strain
3	25.9	5	1.2	40%	0.51
5	21.3	5	1.0	63%	1.01
7	17.5	3.2	0.8	77%	1.48
13	9.6	1.6	0.2	92%	2.55

**Table 2:** FEM simulation parameters of pure Al.

Simulation Parameters	
Material	AA1100 (99% wt.%)
Total number of elements	32,000
The number of steps (step)	60
Step length (sec/step)	0.05
Billet size (mm)	Φ35 × 100
Rolling temperature (°C)	25
Rolling speed (m/s)	0.2
Friction coefficient between billet and groove	0.35
Thermal exchange coefficient between rolls and Al (N/s/mm/°C)	11
Thermal exchange coefficient between air and Al (N/s/mm/°C)	0.016

**Table 3:** Mechanical properties of as-cast and multi-pass caliber rolled pure Al.

Pure Al	TYS (MPa)	UTS (MPa)	El. (%)
as-cast	32	53	35
3P	82	89	19
5P	108	116	15
7P	116	126	15
13P	115	136	17

**Table 4:** Comparison of mechanical properties of pure Al and Al alloy fabricated by various SPD methods.

Alloys	Process	TYS (MPa)	UTS (MPa)	El. (%)	Ref.
Al 1050	RS ^1^, RT, φ ^2^ = 0	20	72	12	[14]
	RS, RT, φ = 0.4	87	91	18	[14]
	RS, RT, φ = 0.8	111	112	15.1	[14]
	RS, RT, φ = 2	137	139	12.9	[14]
	RS, RT, φ = 3	158	163	11.6	[14]
Pure Al	ARB ^3^, 350 °C, 6-cycle	105	113	15	[15]
	ARB, 350 °C, 6-cycle; AN ^4^, 175 °C, 0.5 h	98	109	10	[15]
	ARB, 350 °C, 6-cycle; AN, 175 °C, 6 h	88	93	12	[15]
	E ^5^, 350 °C, 25:1; air cold	146	158	14	[16]
	E, 300 °C, 25:1; water cold	158	158	13.7	[16]
	ARB, 200 °C, 1-cycle	170	172	1.5	[31]
	ARB, 200 °C, 2-cycle	185	200	1.5	[31]
	ARB, 200 °C, 3-cycle	187	205	1.5	[31]
	ARB, 200 °C, 4-cycle	195	230	1.5	[31]
	ARB, 200 °C, 5-cycle	209	243	1.5	[31]
	ECAP ^6^, 456 °C, 4P	111	145	-	[32]
	CR, RT, 13P	115	136	17	This Study
	T4, 380 °C, 2 h	45	75	25	[33]
	T4, 380 °C, 2 h; HPT ^7^, RT,	145	200	8	[33]
	Cryo-rolling, −196 °C, ε = 0.25	130	170	12	[34]
	Cryo-rolling, −196 °C, ε = 0.5	155	175	16	[34]
	Cryo-rolling, −196 °C, ε = 0.75	168	195	12	[34]
Al-0.4Zr (wt.%)	AG ^8^, 375 °C, 60 h; HPT, RT, γ ^9^ = 6.6	96	118	25	[35]
	AG, 375 °C, 60 h; AG; HPT; AN, 230 °C, 1 h	137	142	5	[35]
	AG, 375 °C, 60 h; AG; HPT; AN, 230 °C, 3 h	145	160	6	[35]

^1^ Rotary Swaging; ^2^ Various deformation degrees; ^3^ Accumulative roll-bonding; ^4^ Annealing; ^5^ Extrusion; ^6^ Equal Channel Angular Pressing; ^7^ High-pressure torsion; ^8^ Aging; ^9^ The true strain.

## Data Availability

Not applicable.
